# Endometriosis subtypes and risk of type 2 diabetes: the Advancing Research on Cardiovascular Health and Endometriosis Study (ARCHES) retrospective cohort study

**DOI:** 10.1007/s00125-026-06828-w

**Published:** 2026-08-06

**Authors:** Maggie Fuzak Nunziato, Bin Yan, Karen C. Schliep, Leslie V. Farland, Michael W. Varner, Jenna R. Krall, Kathryn Rexrode, Hediyeh Baradaran, Benjamin Brown, Jennifer J. Majersik, Jessica Page, Anna Z. Pollack

**Affiliations:** 1 College of Public Health, George Mason University, Fairfax, VA USA; 2 Department of Family and Preventive Medicine, University of Utah, Salt Lake City, UT USA; 3 Department of Epidemiology and Biostatistics and Department of Obstetrics and Gynecology, University of Arizona, Tucson, AZ USA; 4 Department of Obstetrics and Gynecology, University of Utah, Salt Lake City, UT USA; 5 Department of Medicine, Brigham and Women’s Hospital, Boston, MA USA; 6 Department of Radiology, University of Utah, Salt Lake City, UT USA; 7 Department of Neurology, University of Utah, Salt Lake City, UT USA; 8 Division of Maternal–Fetal Medicine, Intermountain Health, Murray, UT USA

**Keywords:** Electronic Health Records, Endometriosis Staging, Endometriosis Typology, Type 2 Diabetes

## Abstract

**Aims/hypothesis:**

Endometriosis is a heterogeneous inflammatory condition that may influence long-term metabolic health. Prior studies have reported largely null associations with type 2 diabetes, potentially missing subtype-specific and subgroup heterogeneity. We investigated the association between endometriosis, including clinically relevant subtypes, and incident type 2 diabetes, and assessed effect modification by menopausal status, BMI and history of gestational diabetes mellitus.

**Methods:**

We assembled a dynamic population-based cohort of 2,939,364 individuals assigned as female at birth from the Utah Population Database (1996–2021). Endometriosis and subtypes were identified using validated ICD-9/10 codes and modelled as time-varying exposures. Incident type 2 diabetes was ascertained using ICD codes. Cox proportional hazards models with calendar time as the time scale were used to estimate HRs and 95% CIs, adjusting for birth year, birth state, race/ethnicity, age and BMI at cohort entry. Stratified analyses evaluated effect modification by menopausal status, BMI and history of gestational diabetes mellitus.

**Results:**

During a mean follow-up duration of 10.8 years, 99,978 women were diagnosed with endometriosis. Endometriosis was associated with a 46% higher risk of type 2 diabetes compared with no endometriosis (aHR 1.46; 95% CI 1.43, 1.50). Risk varied by subtype, with the strongest associations observed for ‘other site’ endometriosis (specified site: aHR 2.67; 95% CI 2.51, 2.84; unspecified site: aHR 2.47; 95% CI 2.35, 2.60). Associations were stronger among premenopausal women than postmenopausal women (aHR 1.55; 95% CI 1.50, 1.60) and those with BMI <30 kg/m^2^ than those in higher BMI categories (aHR 2.39; 95% CI 2.34, 2.44), and were present regardless of history of gestational diabetes mellitus.

**Conclusions/interpretation:**

Endometriosis was associated with increased type 2 diabetes risk, with substantial heterogeneity by subtype and metabolic context. Associations were strongest among individuals who are traditionally considered at lower baseline risk, although these findings should be interpreted cautiously given the potential for residual confounding and detection bias.

**Graphical Abstract:**

**Figure:**
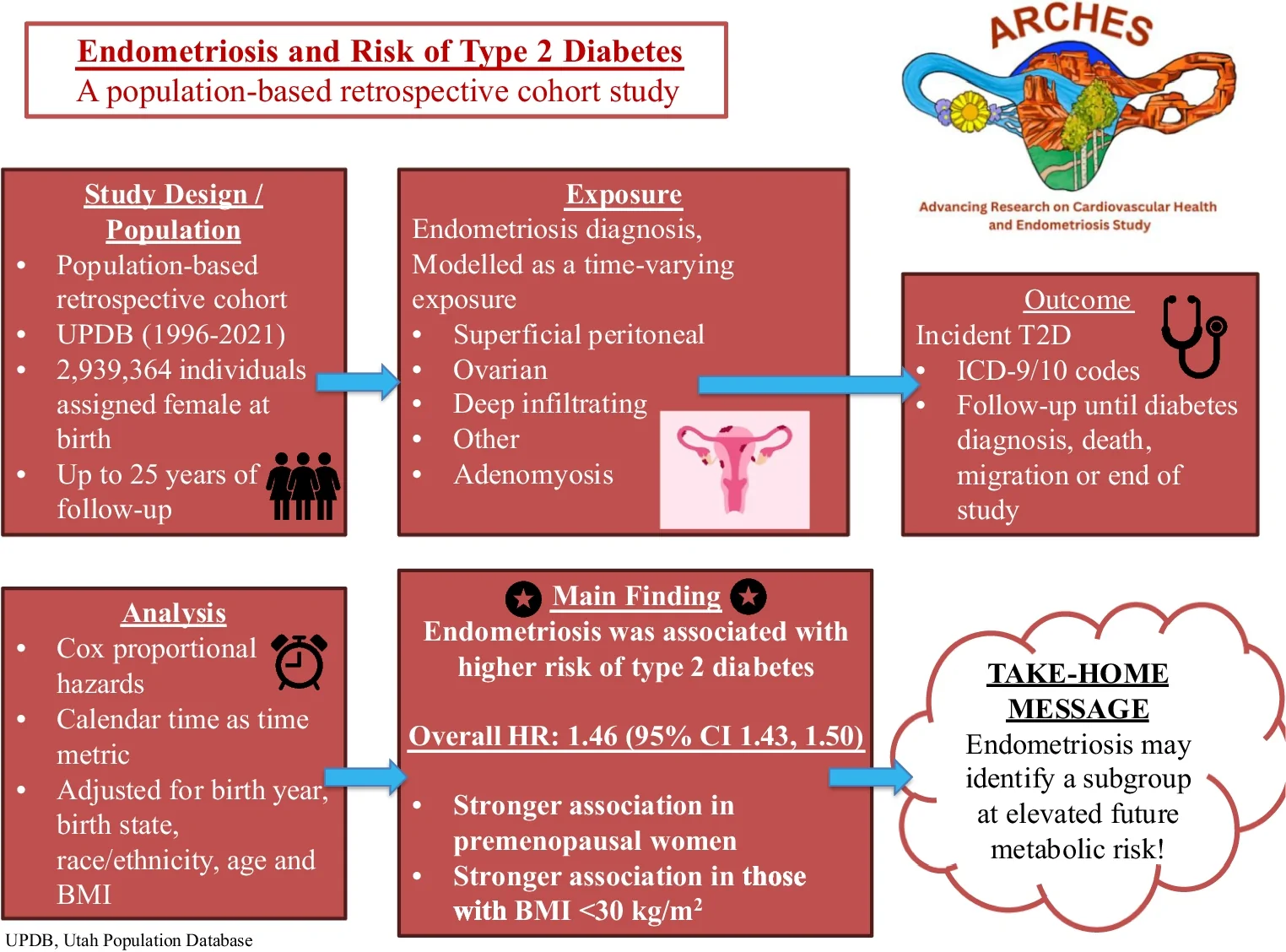

**Supplementary Information:**

The online version contains peer-reviewed but unedited supplementary material available at https://doi.org/10.1007/s00125-026-06828-w.

**Figure:**
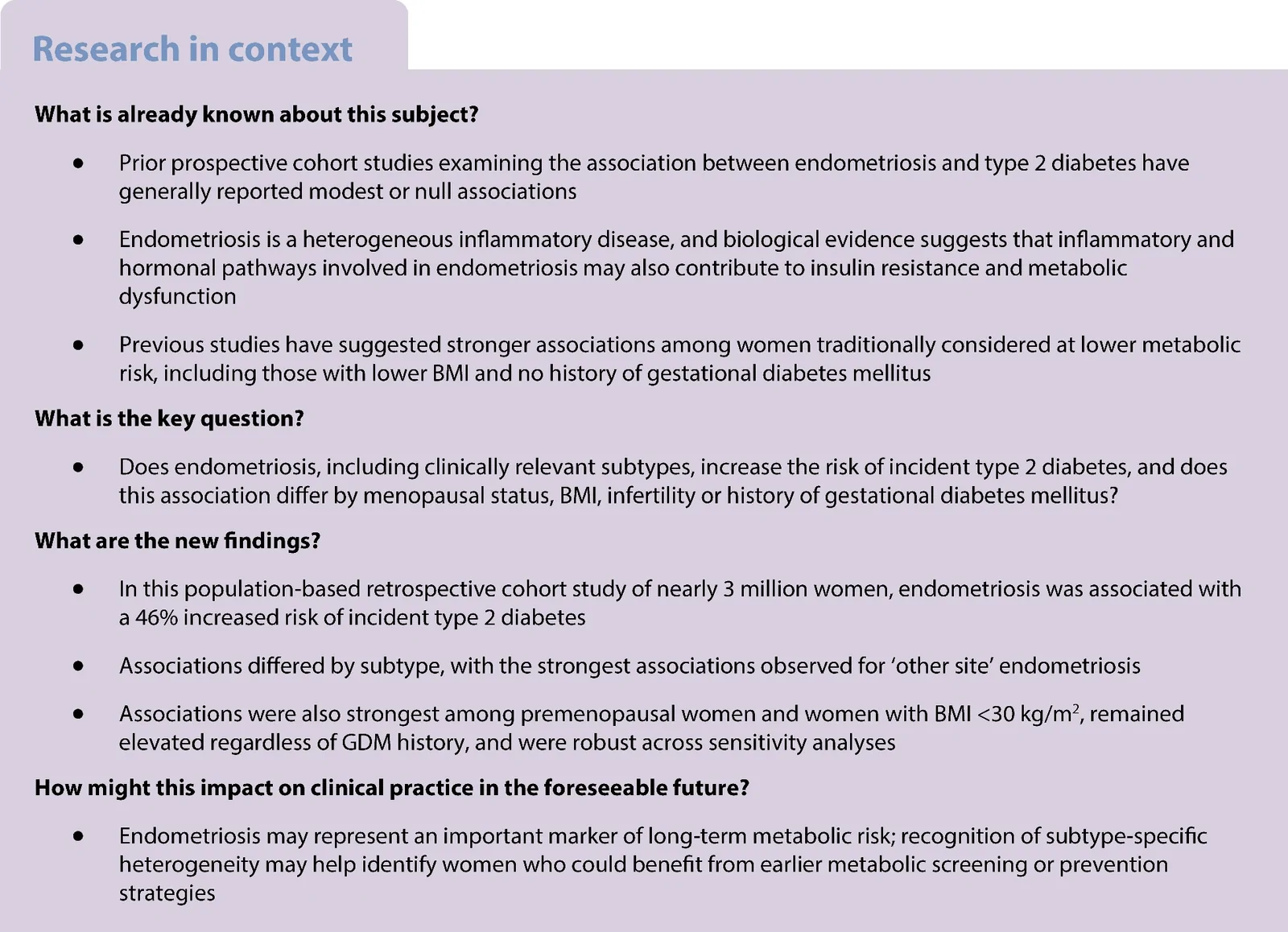

## Introduction

Endometriosis, a chronic gynaecological condition that affects approximately 11% of reproductive-aged women in the USA, is characterised by the presence of endometrial-like tissue outside the uterus [1, 2]. The disease exhibits significant heterogeneity, with symptom presentations ranging from asymptomatic to severe pain and infertility. Ectopic endometrial lesions are classified into three distinct subtypes: superficial peritoneal endometriosis (SE), ovarian endometriosis (OE) and deep infiltrating endometriosis (DE) [3]. SE involves growth of endometrial-like tissue on the peritoneal surface, OE manifests as ovarian cysts (endometriomas) lined with endometrial epithelium, and DE is characterised by endometrial-like tissue infiltrating more than 5 mm beneath the peritoneum, often involving adjacent organs and associated with greater symptom burden [4]. Adenomyosis, which historically has been called ‘endometriosis of the uterus’, is a related but distinct condition that is characterised by the presence of endometrial glands and stroma within the myometrium. Although historically considered a subtype of endometriosis, it is now recognised as a separate disease entity with unique pathophysiology and clinical implications [3, 5].

The heterogeneity of endometriosis subtypes may extend to their systemic effects, including their association with metabolic outcomes such as type 2 diabetes. DE, which is associated with deep tissue invasion and a more severe inflammatory response, may elicit heightened systemic inflammation compared with SE or OE [4, 6]. Inflammation plays a key role in type 2 diabetes pathogenesis, with elevated levels of inflammatory markers such as IL-6 and C-reactive protein having been shown to impair glucose uptake and insulin sensitivity [7]. SE, which also has peritoneal involvement, may also present with an inflammatory profile, while OE, which is often localised to the ovaries, may have unique hormonal and metabolic implications [3, 8]. Previous work has suggested that inflammatory profiles may differ across endometriosis subtypes. For example, IL-6 and other cytokines have been reported to be elevated in the peritoneal fluid of women with ovarian endometrioma compared with those without endometriosis [9] and distinct cytokine signatures have been identified that differentiate SE, OE and DE [10]. Additionally, circulating C-reactive protein levels have been shown to be higher among women with DE compared with control individuals [11]. These findings further support the rationale for examining subtype-specific associations between endometriosis and type 2 diabetes.

Despite these biologically plausible mechanisms that explain the association, findings from large, well-conducted studies have reported modest associations between endometriosis and type 2 diabetes that are not statistically significant [12, 13]. However, subgroup analyses may mask important heterogeneity. For example, the US Nurses’ Health Study II reported a null association between endometriosis and type 2 diabetes (HR 1.09; 95% CI 0.98, 1.13); however, stronger associations emerged among individuals who are traditionally considered ‘low risk’ for type 2 diabetes, such as those with BMI <30 kg/m^2^ (HR 1.17; 95% CI 1.02, 1.35), no history of infertility (HR 1.14; 95% CI 1.04, 1.25) and no prior gestational diabetes mellitus (GDM) (HR 1.11; 95% CI 1.01, 1.22) [12]. In the French E3N cohort, a large prospective study of women with surgically confirmed endometriosis, a null association with type 2 diabetes was reported (HR 1.09; 95% CI 0.92, 1.29), further highlighting potential variability in risk across populations and study designs [13]. Moreover, meta-analyses have reported mixed findings for the association between endometriosis and GDM [14, 15], underscoring the need to explore effect modification by this variable. Assessing factors such as prior GDM history may be key in clarifying the nuanced relationships between endometriosis and future type 2 diabetes risk.

To date, no prior studies have investigated the risk of type 2 diabetes while incorporating information on endometriosis subtype and GDM history. Understanding whether subtype-specific differences modify type 2 diabetes risk could clarify the inconsistencies in the results of prior studies and provide critical insight into the systemic metabolic effects of endometriosis. This knowledge could, in turn, inform targeted interventions, such as personalised screening protocols, early metabolic risk assessments, and tailored lifestyle or pharmacological strategies to mitigate type 2 diabetes risk among individuals with endometriosis, ultimately improving long-term metabolic health outcomes.

## Methods

### Study population

We leveraged the large Utah Population Database (UPDB) to investigate the association between endometriosis and type 2 diabetes. The UPDB is a comprehensive, population-based data resource that includes information on more than 11 million individuals [16]. It uses probabilistic record linking based on multiple identifiers to link vital records (birth, death, marriage), health records from statewide inpatient, ambulatory surgery and emergency department facilities, and University of Utah and Intermountain Health electronic health records, in addition to other records including census information and driver licences (see electronic supplementary material [ESM] Fig. 1) [17]. Our study protocol was approved by the Resource for Genetic and Epidemiologic Research, the University of Utah Institutional Review Board, and the Intermountain Health Institutional Review Board. All research was conducted under a waiver of informed consent approved by the University of Utah Institutional Review Board.

Our UPDB‐based, dynamic cohort comprised all individuals assigned female at birth who were born on 1 January 1935 or later, and who had documented Utah residency on or after 1 January 1996. Cohort entry (‘time zero’) was defined as 1 January 1996 or date of first Utah residency for women who were not residents at the study start date. This approach ensured that all individuals were available for exposure and outcome ascertainment from the start of follow-up, while avoiding inclusion of unobservable person-years prior to Utah residency [18].

### Exposure

Endometriosis exposure was identified using validated [19] ICD-9 and ICD-10 diagnosis codes drawn from all linked health facility sources. In line with prior research [20], we defined endometriosis subtypes by 617* or N80* ICD-9/10 codes: ICD-9 codes 617, 617.2 and 617.3 and ICD-10 codes N80.1 and N80.2 for SE; ICD-9 code 617.1 and ICD-10 code N80.1 for OE; ICD-9 codes 617.4 and 617.5 and ICD-10 codes N80.4 and N80.5 for DE; and ICD-9 codes 617.6, 617.8 and 617.9 and ICD-10 codes N80.6, N80.8 and N80.9, respectively, for other endometriosis including endometriosis in a cutaneous scar, other specified sites (such as bladder, lung or umbilicus), and ‘other site unspecified’. Adenomyosis was defined by ICD-9 code 617.0 and ICD-10 code N80.0. Additionally, in sensitivity analyses, we used laparoscopy codes (ICD-9, 54.21; CPT4 codes 49320–58674; Current Procedural Terminology, 4th edition) to identify women who had been diagnosed with endometriosis using this gold standard of visualised disease [21].

### Outcome

Diagnoses of type 2 diabetes were identified using ICD-9 codes 250.x0 and 250.x2 and ICD-10 codes E11*. In ICD-9, the fifth digits ‘0’ and ‘2’ denote type 2 or unspecified diabetes without and with complications, respectively, thus excluding type 1 diabetes diagnoses. ICD-10 code E11* corresponds specifically to type 2 diabetes mellitus, including related subcodes.

### Covariates

Demographic data, obtained from the UPDB, included birth month and year, birth state (Utah/other state, or born outside the USA), race (American Indian or Alaska Native, Asian, Black or African American, Native Hawaiian or other Pacific Islander, White, multiple races), ethnicity (Hispanic/non-Hispanic) baseline education (less than high school, high school graduate, some college, college graduate and post college), BMI (derived from linked driver licence records), and residence (urban, rural, frontier) [22]. Specifically, frontier areas were defined as highly rural and geographically isolated locations characterised by low population density and substantial travel time to urban centres and essential services, consistent with national and rural health definitions). The Utah Department of Health and Human Services facility provided information on infertility diagnoses and gynaecological surgery (e.g. laparoscopy, laparotomy, hysterectomy, oophorectomy). Birth and foetal death records were used to obtain information on captured parity and pregnancy complications. Death month and year, and last month and year known to be a resident of Utah, were captured from UPDB vital records for censoring purposes.

### Statistical analysis

Women contributed unexposed person-years from cohort entry until their first qualifying endometriosis diagnosis, after which all subsequent person-years were classified as exposed. This time-varying approach avoids the immortal time bias that would arise if women were classified based solely on ever/never endometriosis status at baseline [20]. Follow-up duration accrued from cohort entry until the date of type 2 diabetes diagnosis, death, permanent out-migration from Utah (loss to follow-up), or the end of follow-up (31 December 2021). For time-to-event models, women without type 2 diabetes were administratively censored at their last known date in Utah or 31 December 2021, whichever came first.

Descriptive statistics were used to report characteristics of populations, comparing women with and without endometriosis. Cox proportional hazard models, with calendar time as the time scale, were used to estimate HRs and 95% CIs to assess the risk of developing type 2 diabetes in women with endometriosis compared with those without. We selected calendar time as the time scale to allow us to account for temporal trends in both endometriosis ascertainment and type 2 diabetes diagnosis, which is particularly important in the UPDB given changes in diagnostic practices and healthcare utilisation over time. Consistent with current methodological guidance, departures from the proportional hazards model were not considered a violation of the validity of our model, as Cox model HRs represent weighted averages of time-varying effect after follow-up [23].

Separate models were fitted for SE, DE, OE, DE and OE combined (as both include deep lesions), adenomyosis and ‘other site’ endometriosis, with no endometriosis as the reference group. Models were adjusted a priori for potential confounders including birth year, birth state (born in Utah and not born in Utah), race (American Indian or Alaska Native, Asian, Native Hawaiian or other Pacific Islander, Black or African American, White, multiple races), ethnicity (Hispanic or non-Hispanic), and age and BMI (kg/m^2^) at time of cohort entry (<18.5, 18.5–24.9, 30–34.9, 35–39.9, >40).

Based on prior findings, we tested for effect modification by mean menopausal age (<50 vs ≥50 years), BMI (<30 vs ≥30 kg/m^2^) and history of GDM (yes or no), using stratified models and Wald tests for interaction.

Given the heterogeneity of ICD coding used to define ‘other site’ endometriosis and the potential for diagnostic or healthcare utilisation-related bias, we conducted sensitivity analyses that further refined the ‘other site’ subtype into unspecified and specified anatomical categories. ‘Unspecified other site’ endometriosis was defined using non-specific ICD codes (ICD-9 code 617.9 and ICD-10 code N80.9 or N80.9x), which may reflect incomplete anatomical documentation rather than true disease heterogeneity. ‘Specified other site’ endometriosis was defined by the presence of at least one diagnosis associated with an anatomically specified ICD code (ICD-9 codes 617.6, 617.8 or 617.95 and ICD-10 codes N80.6, N80.8 or N80.Cx); approximately 20% of all cases of ‘other site’ endometriosis were classified as occurring at a specified site.

We conducted several sensitivity analyses to evaluate the robustness of our findings. To assess the impact of the time scale, models were refitted using age as the underlying time scale, with additional adjustment for study start year, with all other covariates unchanged. To address potential delays in endometriosis diagnosis, we conducted analyses assuming that diagnosis occurred 6, 8 and 10 years earlier. We further evaluated the influence of prevalent type 2 diabetes and exposure classification at a time point close to cohort entry by restricting follow-up to 1 January 1998 or later, excluding individuals with type 2 diabetes diagnosed prior to this date while retaining those with endometriosis diagnoses before 1998 and classifying them as exposed.

To assess the potential role of reproductive factors and missing data, we conducted additional analyses adjusting for parity and infertility, performed complete-case analyses restricted to individuals with non-missing covariate data, and incorporated updated electronic health record-derived measures of education, race/ethnicity, smoking and geographic residence. Finally, to evaluate the impact of exposure classification based on surgical confirmation by laparoscopy, we conducted a sensitivity analysis restricted to women with a recorded laparoscopy, for whom endometriosis diagnoses were more likely to reflect surgically evaluated disease; models were otherwise specified identically to the primary analysis.

## Results

Among 2,939,364 women in the UPDB, 99,978 (3.4%) had an incident endometriosis diagnosis during follow-up; the flow chart for cohort assembly is shown in ESM Fig. 2. Compared with women without endometriosis, women with endometriosis were born earlier (median birth year: 1973 [IQR 1962–1982] vs 1986 [IQR 1968–2001]) and were more likely to identify as White (89.3% vs 65.0%). Differences were observed for reproductive and metabolic characteristics: women with endometriosis were substantially less likely to be nulliparous (44.1% vs 77.0%) and more likely to have had one or more births. A greater proportion of women with endometriosis had a healthy BMI (18.5–24.9 kg/m^2^; 56.5% vs 34.8%), and diabetes prevalence was more than twice as high among women with endometriosis than among those without (10.6% vs 4.3%). Women with endometriosis were also more likely to have ever been married (62.4% vs 30.2%), to have a history of smoking (5.2% vs 1.6%) and to reside in urban areas (82.1% vs 80.2%) (Table 1).

**Table 1:** Baseline characteristics of individuals with or without endometriosis from the Utah Population Database, 1996–2021

Characteristic	Total population	No endometriosis	Endometriosis
*n*	2,939,364	2,839,386	99,978
Birth year (median (IQR))	1985 (1968-2001)	1986 (1968–2001)	1973 (1962–1982)
Birth location			
Utah	1,274,234 (43.4)	1,217,767 (42.9)	56,467 (56.5)
Not Utah	1,665,130 (56.6)	1,621,619 (57.1)	43,511 (43.5)
Racial group			
American Indian or Alaska Native	14,385 (0.5)	14,191 (0.5)	194 (0.2)
Asian	37,661 (1.3)	36,785 (1.3)	876 (0.9)
Native Hawaiian or other Pacific Islander	11,022 (0.4)	10,839 (0.4)	183 (0.2)
Black or African American	28,145 (1.0)	27,662 (1.0)	483 (0.5)
White	1,933,900 (65.8)	1,844,647 (65.0)	89,253(89.3)
Multiple races	91,423 (3.1)	87,573 (3.1)	3850 (3.9)
Hispanic			
No	1,747,729 (59.5)	1,665,616 (58.7)	82,113 (82.1)
Yes	363,603 (12.4)	351,691(12.4)	11,912 (11.9)
Maximum educational attainment			
Less than high school	135,649 (4.6)	126,242 (4.4)	9407 (9.4)
High school graduate	318,428 (10.8)	291,094 (10.3)	27,334 (27.3)
Some college	332,719 (11.3)	308,053 (10.8)	24,666 (24.7)
College graduate	166,878 (5.7)	157,570 (5.5)	9308 (9.3)
Post college	67,597 (2.3)	63,657 (2.2)	3940 (3.9)
Marital status			
Ever	920,785 (31.3)	858,391 (30.2)	62,394 (62.4)
Never	1,093,491 (37.2)	1,061,625 (37.4)	31,866 (31.9)
Living place			
Urban	2,358,727 (80.2)	2,276,622 (80.2)	82,105 (82.1)
Rural	466,063 (15.9)	451,705 (15.9)	14,358 (14.4)
Frontier	109,914 (3.7)	106,421 (3.7)	3493 (3.5)
Smoking			
Ever	51,460 (1.8)	46,282 (1.6)	5178 (5.2)
Never	866,066 (29.5)	803,828 (28.31)	62,238 (62.3)
BMI (kg/m^2^)			
<18.5	135,218 (4.6)	128,910 (4.5)	6308 (6.3)
18.5–24.9	1,045,853 (35.6)	989,406 (34.8)	56,447 (56.5)
25–29.9	323,268 (11.0)	304,303 (10.7)	18,965 (19.0)
30–34.9	131,150 (4.5)	123,667 (4.4)	7483 (7.5)
35–39.9	45,440 (1.5)	43,027 (1.5)	2413 (2.4)
>40	30,276 (1.0)	28,704 (1.0)	1572 (1.6)
Diabetes			
Yes	133,733 (4.5)	123,172 (4.3)	10,561 (10.6)
No	2,805,631 (95.5)	2,716,214 (95.7)	89,417 (89.4)

Values are *n* (%) unless otherwise specified

The numbers of missing values were 822,828 (28.0%) for race, 828,032 (28.2%) for ethnicity, 1,918,093 (65.3%) for education, 925,088 (31.5%) for marital status, 4660 (0.2%) for living place, 2,021,838 (68.8%) for smoking, and 1,228,159 (41.8%) for BMI

After adjusting for birth state, birth year, age at cohort entry, race/ethnicity and BMI at cohort entry, women with endometriosis had a 46% higher risk of developing type 2 diabetes (adjusted HR [aHR] 1.46; 95% CI 1.43, 1.50) compared with women without endometriosis (Table 2). To evaluate the robustness of this association to potential unmeasured confounding, we calculated an E-value. The observed HR of 1.46 corresponds to an E-value of 2.28, indicating that the risk ratio for association of an unmeasured confounder with both endometriosis and type 2 diabetes would need to be at least 2.28 to fully explain the observed association. Among endometriosis subtypes, OE was associated with a 61% increased risk of type 2 diabetes (aHR 1.61; 95% CI 1.53, 1.69), while DE was associated with a 45% increased risk (aHR 1.45; 95% CI 1.24, 1.69). Women diagnosed with both DE and OE had an elevated risk (aHR 1.59, 95% CI 1.52, 1.66), and SE was associated with a 67% increased risk of type 2 diabetes (aHR 1.67, 95% CI 1.61, 1.74).

**Table 2:** Associations between endometriosis subtypes and type 2 diabetes within the Utah Population Database, 1996–2021

Variable	Type 2 diabetes cases/person-years	Unadjusted HR (95% CI)	Age-adjusted HR (95% CI)	Multivariable-adjusted HR (95% CI)
Endometriosis				
No	126,142/31,579,954	1.00 (ref)		
Yes	7591/1,047,662	1.68 (1.64, 1.72)	1.42 (1.39, 1.46)	1.46 (1.43, 1.50)
Endometriosis subtype^a^				
No	126,142/31,579,954	1.00 (ref)		
OE	1778/297,861	1.39 (1.33, 1.46)	1.44 (1.38, 1.51)	1.61 (1.53, 1.69)
DE	184/32,607	1.27 (1.09, 1.49)	1.29 (1.10, 1.50)	1.45 (1.24, 1.69)
Combined DE and OE	96/19,890	1.38 (1.32, 1.45)	1.43 (1.46, 1.49)	1.59 (1.52, 1.66)
SE	2876/519,779	1.28 (1.24, 1.33)	1.47 (1.42, 1.53)	1.67 (1.61, 1.74)
Unspecified other site^b^	1481/204,292	1.85 (1.76, 1.95)	2.24 (2.13, 2.35)	2.47 (2.35, 2.60)
Specified other site^c^	235/30,987	1.94 (1.71, 2.21)	2.13 (1.87, 2.42)	2.67 (2.51, 2.84)
Adenomyosis	4348/487,614	2.12 (2.06, 2.19)	1.37 (1.33,1.42)	1.34 (1.30, 1.38)

Multivariable-adjusted models were adjusted for birth state, birth year, race/ethnicity, and age and BMI at time of cohort entry. Calendar time (years from endometriosis diagnosis) is the time metric

^a^Subtype-specific HRs were estimated using separate Cox models. For each model, women without endometriosis and women with other subtypes were excluded

^b^ICD-9 code 617.9; ICD-10 code N80.9 (including N80.9x subcodes)

^c^ICD-9 codes 617.6, 617.8, and 617.95; ICD-10 codes N80.6, N80.8 and N80.Cx

When examining ‘other site endometriosis’ subtypes, both unspecified and anatomically specified diagnoses were strongly associated with type 2 diabetes risk. Women with unspecified ‘other site’ endometriosis had a 2.47-fold higher risk of type 2 diabetes (aHR 2.47; 95% CI 2.35, 2.60), while women with anatomically specified ‘other site’ endometriosis exhibited the strongest association overall (aHR 2.67; 95% CI 2.51, 2.84). Adenomyosis was also associated with an increased risk of type 2 diabetes (aHR 1.34; 95% CI 1.30, 1.38) (Table 2).

We evaluated whether the association between endometriosis and type 2 diabetes differed across key biologically relevant subgroups using stratified Cox models (Table 3). We observed clear heterogeneity by menopausal status (*p* for heterogeneity <0.001). Among premenopausal women (<50 years), endometriosis was associated with a 55% higher risk of type 2 diabetes (aHR 1.55; 95% CI 1.50, 1.60). Among postmenopausal (≥50 years), the association was attenuated and was statistically significant (aHR 1.07; 95% CI 1.07, 1.13). Stratification by BMI also suggested differences in the magnitude of association (*p* for heterogeneity <0.001). Among women with a BMI <30 kg/m^2^, endometriosis was associated with a more than twofold increased risk of type 2 diabetes (aHR 2.39; 95% CI 2.34, 2.44). Among women with BMI ≥30 kg/m^2^, the association attenuated but the risk was still elevated (aHR 1.74, 95% CI 1.70, 1.79).

**Table 3:** Associations between endometriosis and type 2 diabetes, stratified by menopausal status, BMI and history of gestational diabetes, within the Utah Population Database, 1996–2021

Variable	Cases/person-years	Adjusted HR for T2D (95% CI)
Age^a^		
<50 years		
No endometriosis	41,088/25,769,524	1.00 (ref)
Endometriosis	3739/725,803	1.55 (1.50, 1.60)
≥50 years		
No endometriosis	41,668/2,150,614	1.00 (ref)
Endometriosis	1298/105,335	1.07 (1.07, 1.13)
BMI		
<30 kg/m^2^		
No endometriosis	58,055/285,239,127	1.00 (ref)
Endometriosis	10,656/21,450,079	2.39 (2.34, 2.44)
≥30 kg/m^2^		
No endometriosis	43,423/33,837,121	1.00 (ref)
Endometriosis	5855/2,589,089	1.74 (1.70, 1.79)
Gestational diabetes^b^		
No GDM		
No endometriosis	65,904/194,081,406	1.00 (ref)
Endometriosis	12,139/18,222,507	1.99 (1.95, 2.02)
GDM		
No endometriosis	670/1,772,102	1.00 (ref)
Endometriosis	68/168 100,943	2.05 (1.58, 2.66)

Multivariable-adjusted models are adjusted for birth state, birth year, race/ethnicity, and age and BMI at time of cohort entry. Calendar time (years from endometriosis diagnosis) is the time metric

^a^Menopausal status was defined using an age-based proxy, with premenopausal women classified as <50 years of age and postmenopausal women as ≥50 years of age at cohort entry, as clinical menopause is not reliably captured in the UPDB

^b^Analyses stratified by gestational diabetes were restricted to parous women identified from the birth records dataset

T2D, type 2 diabetes

Among parous women with available birth record data, we observed differences by GDM history (*p* for heterogeneity <0.001). Endometriosis was associated with an approximately twofold increased risk of type 2 diabetes among women without a history of GDM (aHR 1.99; 95% CI 1.95, 2.02). A similarly elevated association was observed among women with prior GDM (aHR 2.05, 95% CI 1.58, 2.66) (Table 3).

We conducted several sensitivity analyses to evaluate the robustness of our findings (ESM Tables 1–6). When models were refitted using age as the underlying time scale, the results were consistent in direction with those of the primary analysis, although modestly attenuated; endometriosis remained associated with an increased risk of type 2 diabetes (aHR 1.27; 95% CI 1.24–1.30; ESM Table 1).

To address potential diagnostic delay in endometriosis, we pre-dated the timing of diagnosis by 6, 8 and 10 years (ESM Table 2). Across all lagged exposure definitions, estimates were highly consistent with those in the primary analysis. For overall endometriosis, the aHRs were 1.48 (95% CI 1.45, 1.51), 1.47 (95% CI 1.44, 1.50) and 1.46 (95% CI 1.43, 1.49) for the 6, 8 and 10 year lags, respectively. Subtype-specific estimates followed similar patterns, with the strongest associations consistently observed for ‘other site’ endometriosis (aHRs ranging from 1.96 to 2.00 across lag periods), suggesting that diagnostic delay did not substantially influence the observed associations.

In analyses that restricted follow-up to 1 January 1998 or later, which excluded individuals with type 2 diabetes diagnosed prior to this date while retaining those with endometriosis diagnoses before 1998, associations were modestly attenuated but remained elevated (aHR 1.35; 95% CI 1.32–1.38), indicating that findings were not driven by prevalent type 2 diabetes cases near cohort entry (ESM Table 3).

Additional adjustment for parity and infertility resulted in modest attenuation of effect estimates but did not materially alter the magnitude or pattern of associations across endometriosis subtypes (ESM Table 4). The aHR for overall endometriosis was 1.44 (95% CI 1.41, 1.48), with subtype-specific estimates remaining elevated, including those for OE (aHR 1.55; 95% CI 1.47, 1.62), DE (aHR 1.40; 95% CI 1.20, 1.64), combined DE and OE (aHR 1.53; 95% CI 1.46, 1.60), superficial endometriosis (aHR 1.61; 95% CI 1.55, 1.67), ‘other site’ endometriosis (aHR 1.92; 95% CI 1.83, 2.01) and adenomyosis (aHR 1.34; 95% CI 1.30, 1.38). Complete-case analyses gave similar findings, with an overall aHR of 1.37 (95% CI 1.34, 1.40) and subtype-specific estimates ranging from 1.28 to 1.81.

In sensitivity analyses incorporating updated electronic health record-derived covariates, including education, smoking and urban/rural residence, results were highly consistent with those of the primary analysis (ESM Table 5). The aHR for overall endometriosis was 1.44 (95% CI 1.40, 1.47), and subtype-specific estimates remained similar in magnitude and pattern, including those for OE (aHR 1.60; 95% CI 1.52, 1.68), DE (aHR 1.42; 95% CI 1.22, 1.66), combined DE and OE (aHR 1.58; 95% CI 1.51, 1.65), SE (aHR 1.63; 95% CI 1.57, 1.70), ‘other site’ endometriosis (aHR 1.91; 95% CI 1.82, 2.01) and adenomyosis (aHR 1.32; 95% CI 1.28, 1.36).

Lastly, in sensitivity analyses that restricted the exposed group to women with endometriosis and a recorded laparoscopy confirmation, the association between endometriosis and type 2 diabetes remained elevated but was modestly attenuated compared with the primary analysis (aHR 1.31; 95% CI 1.20, 1.42) (ESM Table 6).

## Discussion

In our large population-based cohort, drawn from the Utah Population Database, we observed that individuals with endometriosis had a substantially higher risk of developing type 2 diabetes than those without endometriosis. In multivariable-adjusted models, endometriosis was associated with a 46% higher risk of type 2 diabetes. Although prior cohort studies [12] have largely reported no overall association between endometriosis and type 2 diabetes, our findings indicate heterogeneity by disease subtype and by individual metabolic risk profiles, showing patterns that may help reconcile inconsistencies in the literature.

Among endometriosis subtypes, the strongest associations with type 2 diabetes were observed for ‘other site’ endometriosis, particularly among women with anatomically specified diagnoses. These individuals experienced nearly a threefold increased risk of type 2 diabetes, while those with unspecified ‘other site’ diagnoses also demonstrated substantially elevated risk. ‘Other site endometriosis’ includes lesions in extra-pelvic or atypical anatomical locations, such as the gastrointestinal tract, abdominal wall or thoracic cavity, and may reflect more anatomically complex or disseminated disease [24]. One potential explanation for the observed associations is the role of systemic inflammation [23, 24]. Endometriosis is characterised by chronic inflammatory activity, and inflammatory pathways have been implicated in the development of insulin resistance and type 2 diabetes [25–27]. However, as our study did not directly measure inflammatory biomarkers, the possible involvement of these mechanisms remains speculative and should be interpreted cautiously. Future studies integrating biomarker data will be important for clarifying the biological pathways underlying the observed associations.

We also observed heterogeneity in the association between endometriosis and type 2 diabetes across clinically relevant subgroups. The association was strongest among premenopausal individuals, with no association observed among postmenopausal individuals. This pattern is biologically plausible, as endometriosis is a hormonally responsive condition that is characterised by heightened inflammatory and proliferative activity during the reproductive years, which may exacerbate metabolic dysfunction prior to menopause [28–31]. In contrast, after menopause, competing age-related metabolic processes may attenuate this relationship. Similarly, associations were stronger among women with BMI <30 kg/m^2^ than among those with BMI ≥30 kg/m^2^, consistent with findings from the Nurses’ Health Study II [12]. One potential explanation is that, among lean women, endometriosis-related inflammation may represent a larger contributor to metabolic dysfunction, whereas in women with obesity, excess adiposity may be the dominant contributor to diabetes risk.

Differences by GDM history further help to explain the metabolic implications of endometriosis. Among parous individuals, endometriosis was associated with elevated type 2 diabetes risk regardless of GDM history, suggesting that pregnancy-related dysglycaemia does not fully explain the observed relationship. Among women with prior GDM, endometriosis may further increase metabolic vulnerability, while the elevated risk among women without GDM indicates that endometriosis-related pathways probably extend beyond traditional mechanisms of insulin resistance.

Several sensitivity analyses supported the robustness of our findings. The results were consistent in direction across the alternative modelling approaches, including use of age as the underlying time scale, although estimates were modestly attenuated. Analyses accounting for diagnostic delay, those that excluded prevalent type 2 diabetes cases near cohort entry, and those incorporating additional non-missing covariate adjustment produced similar patterns of association. Together, these findings suggest that the observed relationship between endometriosis and type 2 diabetes is unlikely to be explained by modelling choices, delayed diagnosis or residual confounding due to measured covariates.

This study has several strengths. The UPDB is an enormous, longitudinal resource with extensive follow-up, allowing for estimation of type 2 diabetes risk over time. The use of time-varying exposure classification minimises immortal time bias and allows for accurate attribution of person-years. Additionally, the ability to evaluate endometriosis subtypes and clinically relevant modifiers, including BMI, menopausal status and GDM, represents an important contribution to the literature.

However, several limitations should be considered. Most endometriosis diagnoses were based on ICD codes, and may be subject to misclassification, particularly for subtype definitions. Although validation studies within this data source demonstrated strong agreement for overall endometriosis diagnosis and for SE and OE, the sensitivity was lower for DE [19]. The ‘other site’ category, which showed the strongest associations, is particularly heterogeneous and may reflect a combination of complex disease and non-specific coding. While sensitivity analyses separating specified and unspecified codes had consistent results, these findings do not fully exclude the possibility of differential healthcare utilisation bias.

Surveillance bias is a key concern in this context. Individuals with endometriosis are more likely to have frequent interactions with the healthcare system, which may increase the likelihood of detection of type 2 diabetes, particularly given that type 2 diabetes can remain asymptomatic for extended periods. Although we attempted to address this through sensitivity analyses, including restriction of prevalent type 2 diabetes cases, the potential for residual detection remains. In sensitivity analyses restricted to women with a recorded laparoscopy, the association between endometriosis and type 2 diabetes was attenuated toward the null. This probably indicates both improved exposure ascertainment and differences in healthcare utilisation. The laparoscopic subset represents a more rigorously evaluated population in which endometriosis diagnoses are less subject to misclassification. However, this group is also highly selected, consisting of women undergoing surgical evaluation, who may differ from the broader cohort in terms of symptom severity, access to care, and healthcare engagement. As a result, these analyses may reduce exposure misclassification but introduce selection bias and limit generalisability, and should therefore be interpreted with caution.

Residual confounding by unmeasured factors, including diet, physical activity and use of hormonal treatments, is also possible. Although hormonal therapies for endometriosis may influence metabolic outcomes, these were not considered in the present analysis. However, E-value analyses suggest that a relatively strong unmeasured confounder would be required to fully explain the observed association. Additionally, sensitivity analyses incorporating more complete covariate data resulted in minimal attenuation of effect estimates. Missing data for several covariates, particularly socioeconomic indicators, may also introduce selection related to healthcare access and data availability, although complete-case analyses resulted in similar findings.

Finally, because the subtype-specific models excluded other endometriosis subtypes from the reference group, HR estimates are not directly comparable across subtypes and should be interpreted within each model context. Additionally, given the multiple outcomes and subtype-specific analyses conducted, there is an increased risk of type I error, and findings should be interpreted with appropriate caution.

In conclusion, our findings suggest that endometriosis is associated with an increased risk of type 2 diabetes, with meaningful heterogeneity across subtypes and metabolic subgroups. Associations were strongest among women who are traditionally considered at lower baseline metabolic risk, suggesting that the association between endometriosis and type 2 diabetes may differ across clinically relevant subgroups. These findings may have implications for future research on metabolic risk assessment among individuals with endometriosis, and support the need for further investigation into the biological mechanisms linking reproductive and metabolic health.

## Supplementary Information

Below is the link to the electronic supplementary material.

Supplementary file1 (PDF 452 KB)

## Data Availability

This study used data from the Utah Population Database (UPDB). The data are subject to strict privacy and regulatory protections and cannot be made publicly available. Qualified researchers may request access through the UPDB Resource for Genetic and Epidemiologic Research (RGE), subject to review and approval by the University of Utah Institutional Review Board and applicable data use agreements.

## Abbreviations

aHR: Adjusted hazard ratio
DE: Deep infiltrating endometriosis
GDM: Gestational diabetes mellitus
OE: Ovarian endometriosis
SE: Superficial peritoneal endometriosis
UPDB: Utah Population Database
