# Correction: Endometriosis subtypes and risk of type 2 diabetes: the Advancing Research on Cardiovascular Health and Endometriosis Study (ARCHES) retrospective cohort study

**DOI:** 10.1007/s00125-026-06864-6

**Published:** 2026-09-08

**Authors:** Maggie Fuzak Nunziato, Bin Yan, Karen C. Schliep, Leslie V. Farland, Michael W. Varner, Jenna R. Krall, Kathryn Rexrode, Hediyeh Baradaran, Benjamin Brown, Jennifer J. Majersik, Jessica Page, Anna Z. Pollack

**Affiliations:** 1 College of Public Health, George Mason University, Fairfax, VA USA; 2 Department of Family and Preventive Medicine, University of Utah, Salt Lake City, UT USA; 3 Department of Epidemiology and Biostatistics and Department of Obstetrics and Gynecology, University of Arizona, Tucson, AZ USA; 4 Department of Obstetrics and Gynecology, University of Utah, Salt Lake City, UT USA; 5 Department of Medicine, Brigham and Women’s Hospital, Boston, MA USA; 6 Department of Radiology, University of Utah, Salt Lake City, UT USA; 7 Department of Neurology, University of Utah, Salt Lake City, UT USA; 8 Division of Maternal–Fetal Medicine, Intermountain Health, Murray, UT USA

**Correction: Diabetologia**

**https://doi.org/10.1007/s00125-026-06828-w**

In the original online version of this article, the adjusted HR for type 2 diabetes for the postmenopausal (≥50 years) women with endometriosis was incorrectly reported as 1.01 (95% CI 1.07, 1.13) rather than 1.07 (95% CI 1.07, 1.13) in Table 3 and in the Results section.

The original article was corrected.

