# Supplementary material for: Endometriosis subtypes and risk of type 2 diabetes: the Advancing Research on Cardiovascular Health and Endometriosis Study (ARCHES) retrospective cohort study
Source: Diabetologia. 2026 Aug 6;69(12):3570–9. doi: 10.1007/s00125-026-06828-w (PMC13714010; doi:10.1007/s00125-026-06828-w)

ESM Tables:

ESM Table 1: Sensitivity analysis using age as the time scale

	Multivariable-adjusted HR (95% CI) – Calendar time (primary model)	Multivariable-adjusted HR (95% CI) – Age as time scale
**Overall Endometriosis**	1.46 (1.43, 1.50)	1.27 (1.24, 1.30)

Age-as-time models used age as the underlying time scale and were adjusted for study start year, birth state, race/ethnicity, and BMI. Models were otherwise specified identically to the primary analysis.

ESM Table 2. Sensitivity analyses pre-dating endometriosis diagnosis by 6, 8, and 10 years

Predated by years	Endometriosis Subtype / Overall	Multivariable-adjusted HR (95% CI)
6	Overall endometriosis	1.48 (1.45, 1.51)
6	Any OE	1.62 (1.55, 1.69)
6	Any DE	1.50 (1.30, 1.72)
6	Any DE and OE	1.60 (1.54, 1.67)
6	Any SE	1.67 (1.61, 1.73)
6	Other site	2.00 (1.92, 2.09)
6	Adenomyosis	1.38 (1.34, 1.42)
8	Overall endometriosis	1.47 (1.44, 1.50)
8	Any OE	1.60 (1.54, 1.67)
8	Any DE	1.54 (1.34, 1.75)
8	Any DE and OE	1.60 (1.53, 1.66)
8	Any SE	1.65 (1.59, 1.70)
8	Other site	1.98 (1.90, 2.06)
8	Adenomyosis	1.37 (1.33, 1.41)
10	Overall endometriosis	1.46 (1.43, 1.49)
10	Any OE	1.59 (1.52, 1.66)
10	Any DE	1.52 (1.34, 1.74)
10	Any DE and OE	1.58 (1.52, 1.65)
10	Any SE	1.63 (1.57, 1.68)
10	Other site	1.96 (1.88, 2.04)
10	Adenomyosis	1.36 (1.32, 1.39)

Endometriosis exposure was redefined in these sensitivity analyses by pre-dating the first recorded diagnosis by 6, 8, and 10 years to account for potential diagnostic delay. Individuals were classified as exposed beginning at the lagged date rather than the recorded diagnosis date. Multivariable-adjusted models are adjusted for birth state, birth year, race/ethnicity, and age, and BMI at time of cohort entry. Calendar time (years from endometriosis diagnosis) is the time metameter. OE, ovarian endometrioma; DE, deep infiltrating endometriosis; SE, superficial peritoneal endometriosis; HR, hazard ratio; CI, confidence interval.

ESM Table 3. Sensitivity analysis excluding T2D diagnoses prior to January 1, 1998

	Multivariable-adjusted HR (95% CI) – Primary model	Multivariable-adjusted HR (95% CI) – Excluding T2D before 1998
**Overall endometriosis**	1.46 (1.43,1.50)	1.35 (1.32, 1.38)

Individuals with T2D diagnosed prior to January 1, 1998 were excluded, while individuals with endometriosis diagnosed before this date were retained and classified as exposed. Multivariable-adjusted models are adjusted for birth state, birth year, race/ethnicity, and age, and BMI at time of cohort entry. Calendar time (years from endometriosis diagnosis) is the time metameter.

ESM Table 4. Sensitivity analyses adjusting for parity, infertility, and complete-case restriction

Exposure / Subtype	Additional adjustment for parity and infertility HR (95% CI)	Complete Case Multivariable-adjusted HR (95% CI)
**Overall endometriosis**	1.44 (1.41, 1.48)	1.37 (1.34,1.40)
**Any OE**	1.55 (1.47, 1.62)	1.47 (1.40,1.54)
**Any DE**	1.40 (1.20, 1.64)	1.32 (1.12,1.55)
**DE + OE**	1.53 (1.46,1.60)	1.45 (1.38,1.52)
**Any SE**	1.61 (1.55,1.67)	1.51 (1.45,1.57)
**Other site**	1.92 (1.83, 2.01)	1.81 (1.73,1.91)
**Adenomyosis**	1.34 (1.30, 1.38)	1.28 (1.24, 1.32)

Multivariable-adjusted models were adjusted for birth state, birth year, race/ethnicity, age at cohort entry, and BMI at cohort entry. Calendar time was used as the underlying time scale. Models evaluating parity and infertility additionally adjusted for parity and infertility. Complete-case analyses were restricted to participants with non-missing values for covariates included in the primary model. OE, ovarian endometrioma; DE, deep infiltrating endometriosis; SE, superficial peritoneal endometriosis; HR, hazard ratio; CI, confidence interval.

ESM Table 5: Sensitivity analysis incorporating updated EHR-derived covariates (education, race/ethnicity, smoking, and urban/rural residence)

Endometriosis / subtype	Multivariable-adjusted HR (95% CI) – Original model	Multivariable-adjusted HR (95% CI) – Updated non-missing covariates
Overall endometriosis	1.46 (1.43, 1.50)	1.44 (1.40, 1.47)
Any OE	1.61 (1.53, 1.69)	1.60 (1.52, 1.68)
Any DE	1.45 (1.24, 1.69)	1.42 (1.22, 1.66)
Any DE and OE	1.59 (1.52, 1.66)	1.58 (1.51, 1.65)
Any SE	1.67 (1.61, 1.74)	1.63 (1.57, 1.70)
Other site	2.47–2.67*	1.91 (1.82, 2.01)
Adenomyosis	1.34 (1.30, 1.38)	1.32 (1.28, 1.36)

Multivariable-adjusted models were adjusted for birth state, birth year, race/ethnicity, age at cohort entry, and BMI at cohort entry. Calendar time was used as the underlying time scale. OE, ovarian endometrioma; DE, deep infiltrating endometriosis; SE, superficial peritoneal endometriosis; HR, hazard ratio; CI, confidence interval.

Updated models additionally incorporated improved EHR-derived measures of education, smoking, race/ethnicity, and geographic residence (urban/rural).
Use of updated EHR-derived covariates reduced missingness for several key variables, including BMI (42% to 31%), ethnicity (28% to 21%), race (28% to 22%), and smoking status (69% to 55%), while education missingness remained largely unchanged (65.3% to 65.0%).

Original “Other site” estimates were presented separately for specified and unspecified categories in the primary analysis

ESM Table 6. Sensitivity analysis restricted to women with laparoscopically confirmed endometriosis

	Multivariable-adjusted HR (95% CI) – Primary model	Multivariable-adjusted HR (95% CI) – Laparoscopy-restricted subset
**Overall endometriosis**	1.46 (1.43,1.50)	1.31 (1.20, 1.42)

Women with endometriosis but without a recorded laparoscopy were excluded from the exposed group, while women without endometriosis remained in the comparison group regardless of laparoscopy status. Women contributed unexposed person-time prior to confirmed endometriosis diagnosis and exposed person-time thereafter. Multivariable-adjusted models were adjusted for birth state, birth year, race/ethnicity, age at cohort entry, and BMI at cohort entry. Calendar time was used as the underlying time scale. HR, hazard ratio; CI, confidence interval.

ESM Figures:

Figure 1: Utah Population Database Linked Records

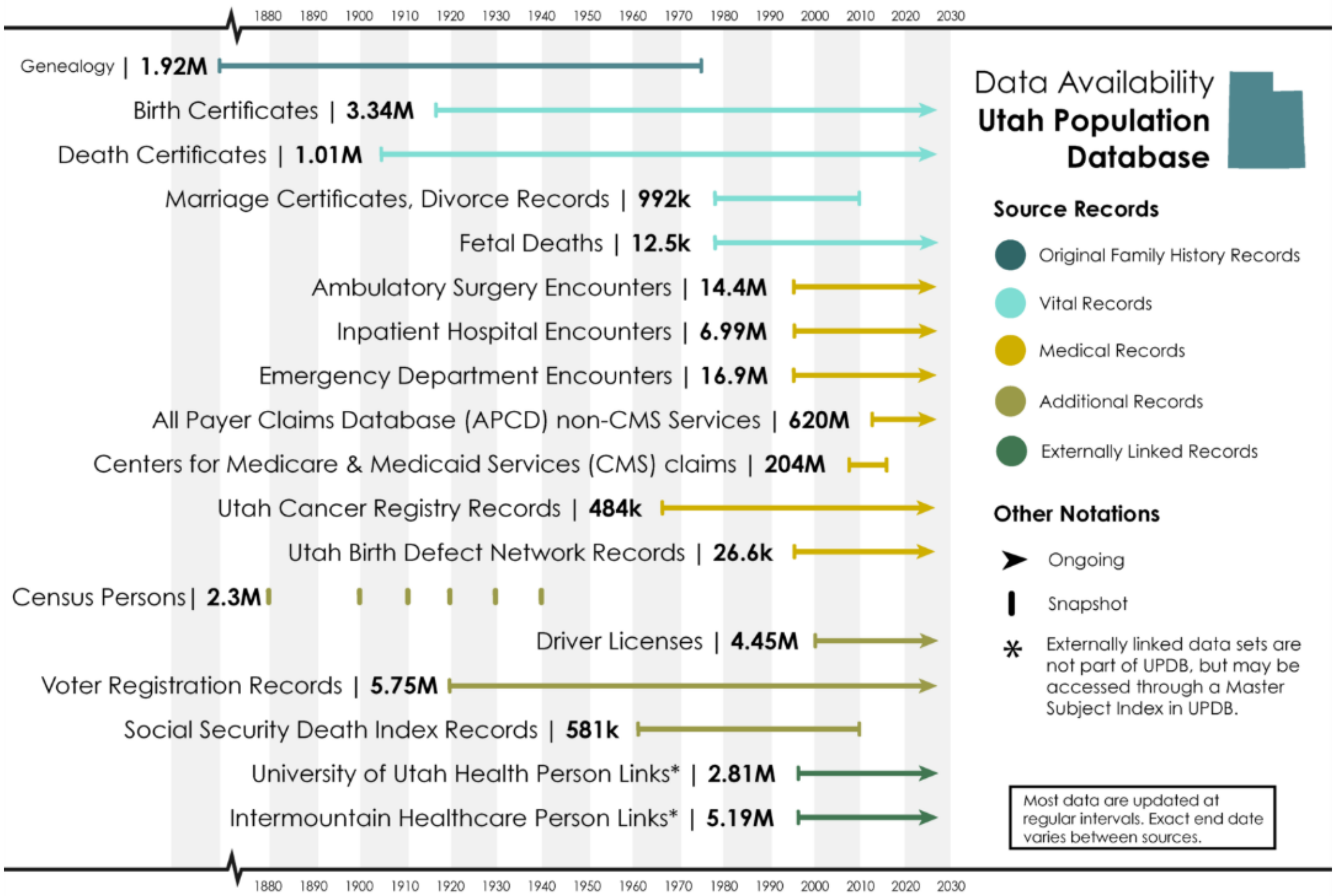

ESM Figure 2: Cohort assembly flow chart for T2D project, Utah Population Database, 1992–2021.

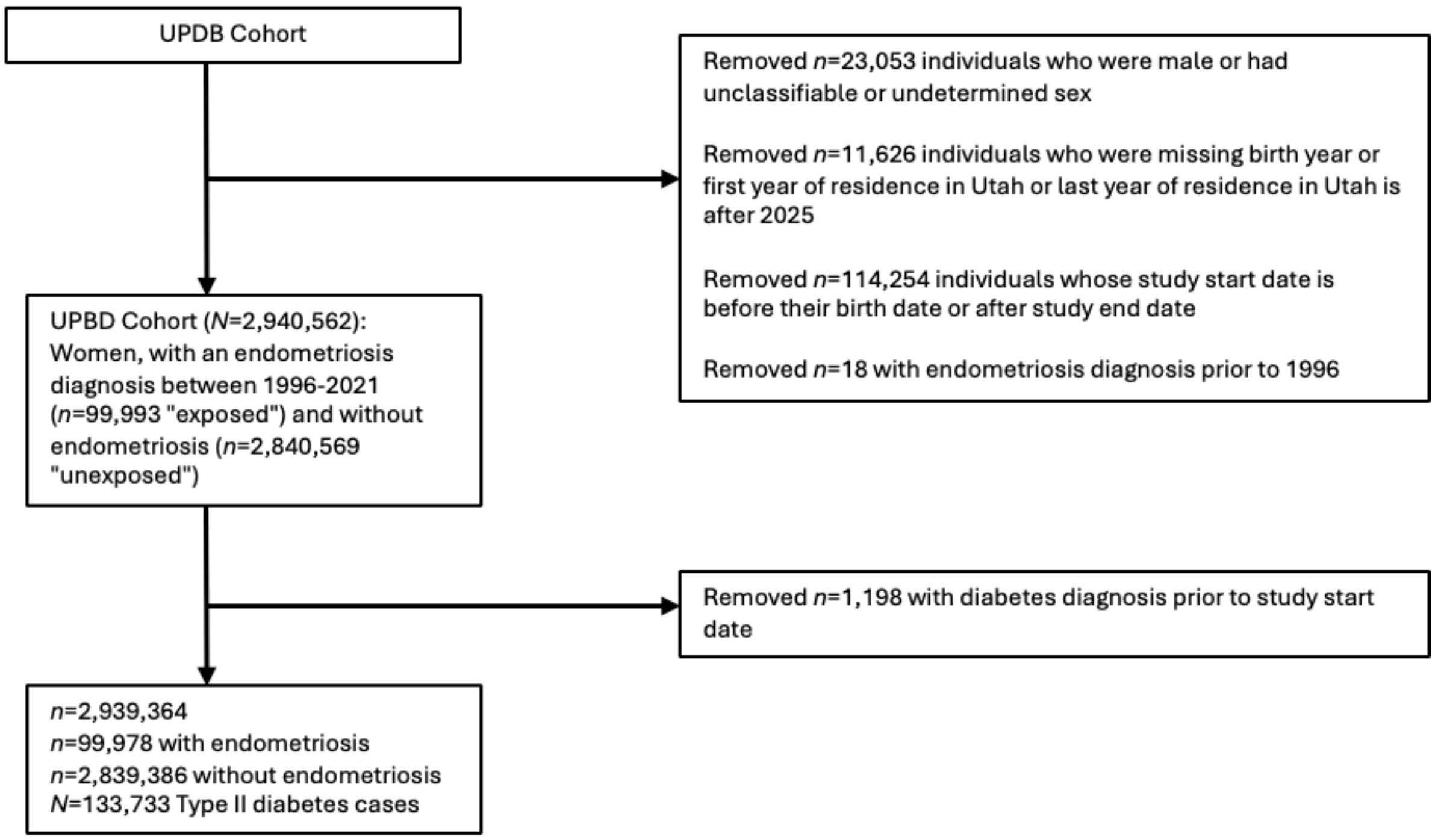

Supplement: Supplementary file 1 [file 125_2026_6828_MOESM1_ESM.pdf]
